# Comparative genomics and transcriptomics of the *Spiroplasma glossinidia* strain *s*Gff reveal insights into host interaction and trypanosome resistance in *Glossina fuscipes fuscipes*

**DOI:** 10.21203/rs.3.rs-7295611/v1

**Published:** 2025-08-25

**Authors:** Daniel J. Bruzzese, Fabian Gstöttenmayer, Brian L. Weiss, Hager Khalil, Robert Mach, Adly M.M. Abd-Alla, Serap Aksoy

**Affiliations:** Yale School of Public Health; Yale School of Public Health; Yale School of Public Health; Joint FAO/IAEA Centre of Nuclear Techniques in Food and Agriculture; Vienna University of Technology; Joint FAO/IAEA Centre of Nuclear Techniques in Food and Agriculture; Yale School of Public Health

**Keywords:** Spiroplasma, Glossina fuscipes fuscipes, tsetse, symbiosis, genome sequencing

## Abstract

Tsetse (*Glossina* spp.) are vectors of African trypanosomes, the causative agents of Human and African Animal trypanosomiases, diseases that remain significant medical and socioeconomic challenges in sub-Saharan Africa. In addition to trypanosomes, tsetse harbor both obligate and facultative symbiotic bacteria that can influence vector competence and reproductive biology. One such facultative symbiont, *Spiroplasma glossinidia*, infects several tsetse species within the *Palpalis* subgroup. In *Glossina fuscipes fuscipes* (*Gff*), the *Spiroplasma glossinidia* strain *s*Gff induces a trypanosome-refractory phenotype and negatively impacts reproductive fitness by reducing female fecundity. However, the mechanisms behind these *Spiroplasma*-derived phenotypes remain poorly understood. Here, we report successful *in vitro* cultivation of *s*Gff and present complete genomes from three sources: *in vitro* cultured *s*Gff and *s*Gff isolated from both laboratory-maintained and wild-caught (Uganda) *Gff* flies. Comparative genomic analyses revealed a high degree of similarity in gene content and synteny among these *s*Gff samples, confirming that they represent isolates of the same strain. Phylogenomic analyses placed *s*Gff within the *Spiroplasma poulsonii* clade. The *s*Gff genome is highly dynamic, containing numerous mobile genetic elements. Additionally, *in silico* annotations indicate that *s*Gff relies on its host for both lipids and carbohydrates and produces several toxins, all of which could be implicated in the observed trypanosome refractory phenotype. Finally, comparative transcriptomic analysis of *s*Gff from host hemolymph versus *in vitro* culture provided insights into potential factors relevant to host-symbiont interactions. Our findings provide a foundation for understanding the nutritional dialogue between *s*Gff and its host and identify symbiotic products that may contribute to trypanosome resistance. Furthermore, the establishment of an *in vitro* culture system for *s*Gff represents a significant resource for future functional studies with potential implications for vector control.

## Background

Tsetse (*Glossina* spp.) transmit African trypanosomes, the causative agents of Human and African Animal Trypanosomiases (HAT and AAT, respectively). Approximately 60 million people in sub-Saharan Africa live in tsetse-infested areas at risk for HAT, while AAT constrains livestock productivity across much of the continent [1, 2]. With no vaccines available for either disease, vector-control strategies remain paramount for disease management [2–4]. Complementary approaches that block or reduce trypanosome development in the fly have the potential to enhance disease control efforts [5]. Successful transmission of trypanosomes is influenced by a combination of intrinsic factors, including the fly’s innate immunity, host and parasite genotypes, as well as host nutritional status at the time of parasite acquisition [6]. In addition to these intrinsic factors, extrinsic factors, including environmental factors and the composition of fly’s microbiota, also modulate pathogen transmission efficiency [5, 7]. In this context, modification of tsetse’s heritable symbiotic microbes, some of which coexist in close proximity to trypanosomes in the midgut, provide a promising avenue for “paratransgenic” interventions [8].

Tsetse species harbor a complex community of heritable symbionts, each playing distinct roles in host biology. All tsetse species carry the obligate mutualist *Wigglesworthia*, which supplements the fly’s nutrient-restricted blood diet with essential vitamins necessary for reproductive success [7, 9], and proper development of the fly’s immune system [10–13]. *Wolbachia* infects tsetse species and can induce cytoplasmic incompatibility, potentially influencing population structure and mating compatibility [14]. *Sodalis* also colonizes tsetse species, with its presence correlated with trypanosome infection prevalence in some contexts, depending on geographic location and tsetse species [15]. Finally, some tsetse house *Spiroplasma glossinidia*, which influences several key tsetse processes, including immune modulation, reproduction, and capacity for trypanosome transmission [16–18]. Despite *Spiroplasma’s* substantial impacts on tsetse biology, the underlying molecular mechanisms governing the bacterium’s interactions with tsetse remain largely unknown.

*Spiroplasma* are helical, wall-less Mollicutes, first characterized as plant pathogens [19, 20], but are now known to colonize a wide range of arthropod hosts, most commonly infecting insects [21–27]. In insects, *Spiroplasma* exhibits a broad range of symbiotic phenotypes, including protective effects in different *Drosophila* species against parasitic wasps and nematodes by either producing ribosome-inactivating proteins (RIPs), which disrupt parasite protein synthesis machinery [28–30], or by competing with the parasite for macronutrients [31]. Conversely, some *Spiroplasma* strains act as reproductive parasites in insects, such as in *Drosophila* [32], *Anisosticta* (ladybugs) [33], and *Danaus* butterflies [34], where they induce selective male-killing. Of note, *Spiroplasma* genomes evolve rapidly, at rates comparable to RNA viruses [35]. This rapid pace of evolution is attributed to the abundance of mobile genetic elements and absence of key DNA mismatch repair genes, which together promote genomic plasticity, rapid diversification, and horizontal gene transfer of these key symbiosis genes [23, 36].

Within *Glossina*, *S. glossinidia* infections are limited to species in the *Palpalis* subgroup, including *Glossina fuscipes fuscipes (Gff*), *Glossina palpalis palpalis*, and *G. tachinoides* [14, 37]. In Uganda, the *S. glossinidia* strain infecting *Gff* (*s*Gff) is geographically restricted and polymorphic, with prevalence ranging from 5–34% in Northwestern populations, while absent from Central and Southern regions. The infection prevalence in Uganda remains relatively stable across time and space, although seasonality can impact infection dynamics [18]. Interestingly, in the *Gff* line reared from the Insect Pest Control laboratory at the International Atomic Energy Agency (IAEA), *s*Gff infection is also not fixed, but is stably maintained at approximately 50% prevalence [38]. Laboratory transmission studies in this *Gff* line indicate that *s*Gff is maternally inherited with high fidelity, although paternal transmission can also occur [17]. The bacterium colonizes multiple tissues, such as the gonads, gut, and hemolymph [14, 16]. *Gff* infected with *s*Gff show altered gene expression in reproductive and gut tissues [16], reduced hemolymph triacylglyceride (TAG) levels, impaired sperm fitness, and reduced female fecundity [17]. In addition, *s*Gff infection is negatively correlated with trypanosome infection prevalence in both field and lab populations, suggesting that presence of the bacterium induces a parasite refractory phenotype in tsetse [18].

Whether *s*Gff directly (e.g., via the production of anti-trypanosomal factors) or indirectly (e.g., competition for nutrients) confers the observed parasite resistance phenotype to its tsetse host remains unknown. Transcriptomic analyses of *s*Gff-infected *Gff* midguts revealed minimal immune stimulation but indicated elevated oxidative stress and impaired lipid biosynthesis [16]. These changes may create a hostile gut environment for parasites by increasing production of trypanocidal nitric oxide (NO) and/or by reducing the availability of key metabolites required for trypanosome survival.

To investigate the mechanisms by which *Spiroplasma* alters tsetse’s physiology and impacts vector competence, we leveraged recently developed *in vitro* methods to culture *s*Gff. We then sequenced whole genomes of *s*Gff from *in vitro* culture, laboratory-reared, and wild-caught *Gff* individuals using the Oxford Nanopore Technology (ONT) platform. Comparative analysis of the three *s*Gff genomes revealed subtle isolate-specific genetic variation residing primarily in mobile genetic elements. We also characterized *s*Gff gene expression from both *in vitro s*Gff and *s*Gff-infected hosts, and identified core metabolic pathways and candidate toxins that could mediate the tsetse-*s*Gff symbiosis. Collectively, this study lays the foundation for dissecting the molecular basis of the *s*Gff–*Gff* interactions and for evaluating its potential in trypanosomiasis control strategies.

## Methods

### Initiation of sGff in vitro culture

Establishment of the *s*Gff *in vitro* culture followed the protocol of Masson et al. [39], developed and optimized for the cultivation of *s*MSRO. This protocol is based on Barbour-Stoenner-Kelly H (BSK-H) media without L-Glutamine (Bio&Sell, Germany), originally designed for *Borrelia burgdorferi,* supplemented with rabbit serum, *Gff* fly extract, lipids, antibiotics, and amino acids. Detailed media and cultivation protocols are indicated in Supplementary Document 1. Briefly, hemolymph from female and male *Gff* from a laboratory line with high *s*Gff infection prevalence [38] was collected by removing one prothoracic leg and aspirating the exposed hemolymph droplet using a 10 μl pipette tip. Cultures were established in three biological replicates using 10 μl hemolymph and incubated at 25°C for 14 days without agitation under microaerobic atmospheric conditions. Routine microscopy checks were performed by fluorescent microscopy on a Leica DMi8 inverted microscope (Leica Microsystems) following staining with SYTO 9 (0.025 mM; ThermoFisher Scientific). *Spiroplasma* presence was further confirmed by PCR-amplification using *Spiroplasma-*specific 16s rRNA primers (Supplementary Table 1). Cultures underwent passaging every 10 – 14 days by diluting cultures 1:1 with fresh media.

### *In vitro* culture growth kinetics

Samples from *s*Gff cultures for growth curve analysis were collected in triplicate biological replicates from day 0 to day 15 following the inoculation of BSK-H-spiro media with *s*Gff precultures. Each day, five μl of culture was diluted in 200 μl nuclease-free double-distilled water (ddH_2_O) in a sterile PCR tube and stored at −20°C. Prior to quantitative PCR (qPCR), samples were lysed via osmotic heat-shock by incubation at 95°C for 10 minutes. qPCR reactions were performed in triplicate technical replicates, in a final volume of 15 μl consisting of 7.5 μl iQ^™^ SYBR^®^ Green Supermix (Bio-Rad, CA, USA), 5.5 μl PCRgrade water, 0.5 μl each of the forward and reverse *Spiroplasma* qPCR primers (Supplementary Table 1), and 1 μl heat-shocked culture. To accurately estimate *Spiroplasma* copy numbers, a standard was prepared by purifying *s*Gff-specific qPCR amplicons using the Zymo DNA Clean & Concentrator-25 kit (Zymo Research, USA), followed by DNA quantification with a nanodrop spectrophotometer. The purified DNA was then serially diluted 1:10 to create a range of known concentrations. Cycling conditions for the qPCR comprised of an initial denaturation step at 95°C for 2 min, followed by 40 cycles of 95°C for 5 sec, and 56°C for 30 sec on a Bio-Rad CFX96 Touch Real-Time PCR Detection System (Hercules, CA, USA). Raw Ct values were extracted in the CFX maestro software and analyzed using R Studio v4.4.2 [40, 41]. Briefly, the mean value of the three technical replicates of each sample was calculated and the copy number of each sample was inferred based on the standard curve of known copy numbers [42]. Calculation of culture doubling time was performed according to the formula *log*(2)*/slope* [43].

### Injections of *in vitro* cultured *sGff* into naïve *Gff*

Teneral females and males from the *Gff* line were placed in individual cages and their *Spiroplasma* infection status was determined by PCR-amplification of DNA extracted from one mesothoracic leg using *Spiroplasma* 16s rRNA and tsetse tubulin gene primers (Supplementary Table 1). Negative-*s*Gff flies were pooled into two treatment groups (12 females and 6 males per group): (1) high dose and (2) low dose. In addition, 12 females and 6 males screened positive for *s*Gff were retained as a control group with a natural infection. *Gff* were injected using 29-gauge insulin needles on a micrometer syringe unit injector (Burkard Manufacturing, UK). Injected copy numbers were estimated via quantitative PCR compared to a standard curve of known copy numbers: High Dose: 1.1 × 10^5^ copies in Replicate 1 and 4.3 × 10^5^ copies in Replicate 2; Low Dose: 5.7 × 10^2^ copies in Replicate 1 and 1.2 × 10^3^ copies in Replicate 2. Three hours post-injection, flies were offered their first blood meal and subsequently maintained under standard rearing conditions [44] for 14 days, receiving defibrinated bovine blood three times a week via an artificial membrane feeding system [45]. At designated time points (0, 1, 6, 9 and 14 days), two females and one male were collected, flash-frozen, and stored at −20°C. Genomic DNA from individual whole flies was extracted using the Qiagen DNeasy Blood & Tissue kit (Qiagen, Hilden, GER) according to the manufacturer’s protocols and diluted to 4 ng/μl. The relative abundance of *s*Gff over time was measured by qPCR, with three technical replicates per sample. Ct values for *sGff* were normalized to those of the tsetse housekeeping gene β-tubulin (Supplementary Table 1) and relative fold-change was calculated using the 2^−(ΔCt)^ method [46]. Statistical analysis was performed in RStudio v4.4.2 [40, 41]. Significant differences in *s*Gff fold-change across timepoints between and within each group were identified using the Kruskal-Wallis test and Dunn’s post-hoc test with Benjamini-Hochberg correction for multiple comparisons.

### sGff sequencing and assembly

Three *Gff Spiroplasma* (*s*Gff) whole genome assemblies were generated: (1) *s*Gff-IAEA-CC - derived from the *in vitro* culture of *Spiroplasma* established in 2023 from IAEA *Gff* females and males as described above; (2) *s*Gff-IAEA-Fly - assembled from a previously sequenced *Spiroplasma-*infected *Gff* female obtained in 2023 from the IAEA colony (NCBI BioSample: SAMN47211303 and NCBI SRA: SRR32737746), and (3) *s*Gff-UG-Tol - assembled from a previously sequenced *Spiroplasma*-infected *Gff* female collected in 2019 from Toloyang village in Atiak Sub County, Amuru District, Uganda (3.304883654, 32.37452634) (NCBI BioSample: SAMN47211302 and NCBI SRA: SRR32737747) [47].

For *s*Gff-IAEA-CC genome, high density *in vitro Spiroplasma* culture (~10^6^ cells) was collected at passage nine, pelleted, and washed with PBS. HMW DNA was extracted using the PureGene kit (Qiagen, Hilden, GER). An ONT LSK14 library was prepared following standard protocols and sequenced on a ONT 10.4.1 minION flow cell for 12 hours. Generated POD5 signal data files were basecalled with Dorado v0.9.1 (https://github.com/nanoporetech/dorado) using the SUP duplex pipeline. Simplex reads were dropped with Samtools v1.18 [48], and remaining sequencing adapters were trimmed using Dorado trim. The produced FASTQ files were quality filtered with nanoq v0.10.0 [49] to have a minimum quality of q10 and minimum 3000 bp read length. To assemble the *s*Gff-IAEA-CC genome, Autocycler v0.2.1 (https://github.com/rrwick/Autocycler) was used to generate multiple assemblies from four independent subsampled 100X read sets using five assemblers for each read set (Flye v2.9.5 [50], Canu v2.3 [51], miniasm v.3 [52], NECAT [53], and NextDenovo v2.5.2 [54]). A consensus assembly for these 20 assemblies was then determined with Autocycler v0.2.1 and polished with Medaka v2.0.1 using the bacteria model (https://github.com/nanoporetech/medaka). The genome was further polished using short reads made from the same HMW DNA used for ONT sequencing. These reads were sequenced on an Illumina NovaSeq X system (Eurofins Genomics, Germany) and quality trimmed to Q30 with a minimum length of 50 bp with fastp v0.24.0 [55]. Short read polishing was performed with Polypolish v0.6.0 [56] followed by pypolca v0.3.1 [57].

To assemble the *s*Gff-IAEA-Fly genome, we mapped raw Nanopore simplex reads generated with the SUP Dorado v0.9.0 model from the *Gff*-IAEA genome assembly (SRR32737746) to the *s*Gff-IAEA-CC assembly using minimap2 v2.28 [58]. Unmapped reads were dropped with Samtools. The remaining simplex reads were precorrected with Dorado correct to generate near–Hifi quality reads and were assembled using six assemblers with varying parameters (Hifiasm v0.25 [59], Flye, Canu, miniasm, NECAT, and NextDenovo). A consensus assembly was determined with Autocycler v0.2.1.

Finally, to assemble the *s*Gff-UG-Tol genome, we mapped raw Nanopore simplex reads generated with the SUP Dorado v0.9.0 model from the *Gff*-IAEA genome assembly (SRR32737747) to the *s*Gff-IAEA-CC assembly using minimap2. Unmapped reads were dropped with Samtools. Due to the shorter read-lengths of this dataset we were unable to utilize Dorado correct to precorrect ONT reads. Instead, we assembled the mapped ONT Simplex reads using five assemblers (Hifiasm (--ont), Flye, miniasm, NECAT, and NextDenovo). A consensus assembly was determined with Autocycler v0.2.1 and was polished with Medaka v2.0.1 using the bacteria model.

All completed *s*Gff circular genomes and plasmids were reoriented to start with the *dnaA* or *repA* with Dnaapler v0.7.0 [60]. Assembly stats for the assembled genomes were established using gfastats v1.3.10 [61] and genome completeness was assessed with BUSCO v5.8.3 [62] using the entomoplasmatales_odb10 dataset. Plasmids were clustered with pling v2 [63] to determine similarity and were named in order according to their size. All genomic data were deposited under the NCBI BioProject: PRJNA1235259.

### Functional annotations

Assembled genomes were first annotated using the NCBI Prokaryotic Genome Annotation Pipeline [64]. For the reference *s*Gff-IAEA-CC genome, additional functional annotations were added with Bakta [65], eggNOG-mapper [66], BlastKOALA [67], and COGclassifier (https://github.com/moshi4/COGclassifier). Mobile genetic elements were annotated using PHASTEST [68] for prophages, ICEberg v3 [69] for integrative conjugative elements, and ISEScan v1.7.3 [70] for insertion sequence elements. Metabolic pathways were characterized with the KEGG terms assigned above with eggNOG-mapper [66] and BlastKOALA [67]. Putative proteins were analyzed using SignalP v6 [71] to determine N-terminal signal peptide sequences, indicative of secretion. Finally, *Spiroplasma* specific symbiosis genes identified from the literature were manually searched for with BLASTP [72] (Supplementary Table 2). To better characterize the RIP identified in *s*Gff, we generated a phylogeny using *Spiroplasma* members of the RIP superfamily from InterPro [73] and our putative RIP sequence (Supplementary Table 3). The tree was constructed using RAxML-NG [74] with the LG+G8+F model, with 25 parsimony and 25 random starting trees with 1000 bootstraps using PRANK [75] aligned amino acid sequences trimmed to only contain RIP domains.

### Spiroplasma phylogenomics

To determine *s*Gff’s relationship to other *Spiroplasma* strains, we constructed phylogenetic trees at two scales: (1) using *Spiroplasma* strains from several clades, including *Spiroplasma citri*, *Spiroplasma mirum, Spiroplasma chrysopicola,* and *Spiroplasma poulsonii*, and (2) using only members from the *S. poulsonii* clade. Reference genomes and their NCBI RefSeq (or if missing, NCBI GenBank) annotations in GBFF format were downloaded using NCBI datasets [76]. These genomes were clustered using RabbitTClust [77], to iteratively drop *Spiroplasma* strains from distant clades (e.g. *Spiroplasma ixodes* or *Spiroplasma apis*). For the remaining *Spiroplasma* strains (Supplementary Table 4), single-copy orthologous core genes (present in > 95%) of taxa were identified with PPanGGOLiN [78]. From this gene set, DNA sequences were extracted, aligned with MAFFT [79], and concatenated for each *Spiroplasma* taxa. Phylogenetic trees were generated from the multiple sequence alignment using RAxML-NG [74] with the GTR+G substitution model with 50 random and 50 parsimony-based starting trees, and with 1,000 bootstrap replicates. The resulting draft RAxML tree was used to identify duplicate *Spiroplasma* strains, which were removed before re-running the PPanGGOLiN and RAxML pipeline. Finally, the whole process was repeated at a smaller scale for members of the *Spiroplasma poulsonii* clade only.

### Genome comparisons

Pairwise average nucleotide identity was calculated between the three assembled *s*Gff genomes and the closest sister taxa, TU-14 (GCF_001792795.1), using pyani v0.2.13.1 [80]. Whole-genome synteny, SNP, and indel comparisons between *s*Gff-IAEA-CC, *s*Gff-IAEA-Fly, and *s*Gff-UG-Tol were performed by pairwise alignments using NUCmer [81] annotated with SyRI [82], and visualized using plotsr v1.1 [83]. For the *s*Gff-IAEA-CC, *s*Gff-IAEA-Fly, *s*Gff-UG-Tol, and sister taxa TU-14 (GCF_001792795.1), we identified shared and unique gene families by parsing the PPanGGOLiN dataset generated above with for the *Spiroplasma poulsonii* RAxML tree using R [40] with the tidyverse [84] and VennDiagram [85] packages. Unique gene family amino acid sequences were functionally annotated with Bakta [86].

### RNA sequencing and analyses

To assess *s*Gff transcriptomes, five *s*Gff RNA samples were prepared for RNAseq: three from *s*Gff cell-culture and two from *Gff* hemolymph. For the *s*Gff cell-culture samples, *s*Gff-IAEA-CC (~10^6^ cells) at passage 12 were pelleted at 12,000g for 15 mins at 4°C and resuspended in RNAlater^™^ (Invitrogen, MA, USA) and stored at −80°C. For the *Gff* hemolymph samples, female IAEA *Gff* flies were screened for *s*Gff infections using the above PCR protocol. Positive flies were surface-sterilized in 70% ethanol and hemolymph was collected as described above. Hemolymph was pooled into two samples from 70 and 35 *s*Gff-positive females and stored in RNAlater^™^ (Invitrogen, MA, USA) at −80°C. Total RNA was extracted from samples by first diluting the samples stored in RNAlater 1:1 with PBS, pelleting at 5000g for 15 mins at 4°C, and resuspending the pellets in 50 μl PBS. Total RNA was extracted from the PBS washed samples using the Total RNA Miniprep kit (NEB, MA, USA) following standard protocols. Ribodepleted RNA-seq libraries were prepared by the Yale Center for Genomic Analysis and sequenced on the NovaSeq 6000 platform. Raw reads were quality filtered (Phred score >20 and read length >50 bp) and adapter-trimmed using fastp v0.24.0 [55]. Trimming and filtering results were summarized with MultiQC v1.27 [87].

We first used the RNA sequencing data to validate the *in silico s*Gff-IAEA-CC annotations. We pooled all transcriptomes into a single sample and quantified transcript abundance using Salmon [88] with the *s*Gff-IAEA-CC genome as a reference. Transcripts were considered to be expressed if their transcripts per million (TPM) count was > 5. Operons were identified with the OpDetect v1.0 [89] pipeline, which utilized STAR v2.7.11b [90] alignments of the transcriptome to the *s*Gff-IAEA-CC genome. We then quantified differential gene expression for the three culture and two *Gff* hemolymph samples, again utilizing Salmon [88] to quantify transcript abundance to the *s*Gff-IAEA-CC reference genome. Differential transcript analysis was performed with edgeR [91] using the quasi-likelihood model. We defined significantly differentially expressed genes as those with a false discovery rate (FDR) < 0.05 and log-fold-change (LFC) > 1.5 or < −1.5.

## Results

### Establishment of *s*Gff *in vitro* cultures

**Figure 1:**
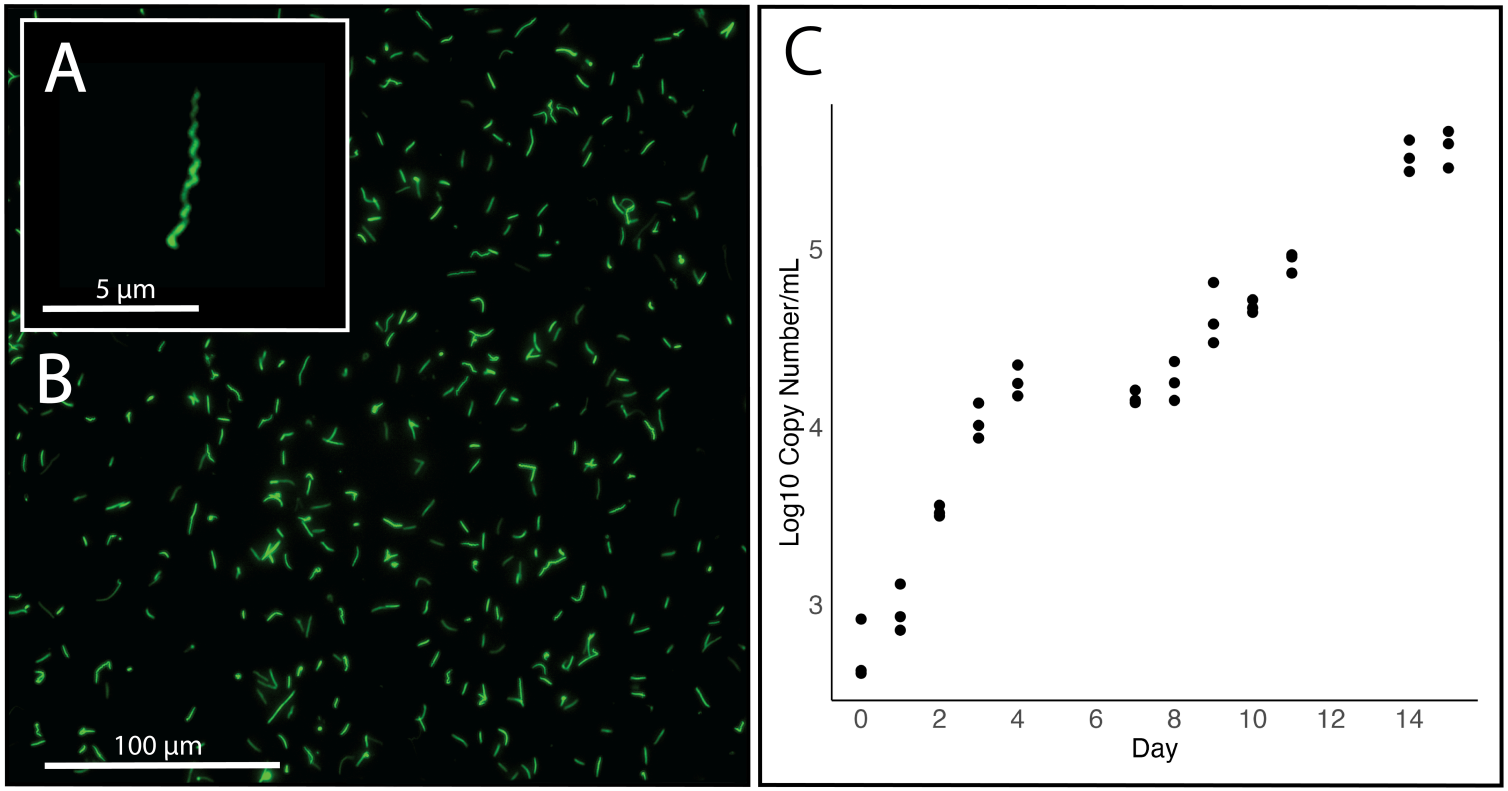
*In vitro* cultivation of *s*Gff. A) Fluorescent microscopy of *in vitro s*Gff cells at passage nine stained with SYTO 9 and imaged using a Leica DMi8 inverted microscope (FITC channel). Scale bar: 5 μm. B) High density *s*Gff culture at passage nine. Scale bar: 100 μm. C) Growth kinetics of*s*Gff in supplemented BSK-H media at 25°C under microaerobic conditions, assessed by qPCR with threefold technical and biological replication.

Following the published protocol for *s*MSRO, we successfully established an *in vitro* culture of the *Spiroplasma* strain *s*Gff isolated from the hemolymph of *Gff*. Microscopic examination of SYTO 9-stained cells revealed a homogenous culture, with cells exhibiting the characteristic helical morphology of *Spiroplasma* (Fig 1A and 1B). Growth curve analysis of cultures inoculated with frozen preculture aliquots identified two distinct phases of exponential growth, occurring from days 0 to 4 and days 7 to 11, separated by a plateau phase (Fig 1C). The estimated doubling time was 40 hours during the first exponential phase and 85 hours during the second. Fluorescence microscopy revealed that cultures reached high cell densities within 14 days post inoculation. Remarkably, the *s*Gff cultures have remained viable for over 12 months with routine subculturing every 10–14 days, demonstrating the robustness and long-term stability of the *in vitro* system.

### *s*Gff cultures can establish infections in naïve *Gff* hosts

To assess whether *in vitro*-cultured *s*Gff retains its ability to replicate *in vivo* following prolonged culture, we conducted microinjection experiments using two groups of naïve *Gff* that were confirmed negative for *s*Gff by PCR screening of a mesothoracic leg. *s*Gff-negative teneral flies were injected with either a high- or low-dose of the cultured *s*Gff and infection dynamics were monitored over a 14-day period. The results demonstrate that *in vitro*-cultured *s*Gff retains its replicative capacity within naïve *Gff*, validating its potential utility for downstream functional studies. Quantification of *s*Gff in transinfected *Gff,* alongside naturally infected *Gff* controls, revealed differences in initial *s*Gff titers between groups on day 0, with the low-dose group showing the lowest titer (fold-change = 0.014), and high-dose and natural infection groups exhibiting similar titers (fold-change = 2.3 and 2.451, respectively) (Supplementary Table 5). We observed a dose-dependent increase within groups over time (Supplementary Figure 1). Statistical analysis confirmed significant differences in *s*Gff titers between treatment groups on days 0, 1 and 6 (Kruskal-Wallis: *p*-values = 0.003, 0.003 and 0.0007, respectively) (Supplementary Table 6) and a significant increase within both high- and low-dose groups over time (Kruskal-Wallis: *p-*value = 0.000044 for low-dose; *p*-value = 0.00045 for high-dose) (Supplementary Table 7), with post-hoc tests indicating that titers on days 9 and 14 were significantly higher compared to days 0 and 1 (Supplementary Tables 8 and 9). In contrast, the increase of *s*Gff titers in the naturally infected control group was not statistically significant, likely due to higher variability among individuals (Supplementary Table 5).

### sGff complete genome assembly

To generate a high-quality reference genome for *s*Gff, we sequenced the whole genome and obtained 78,639 Nanopore duplex reads (565.8 Mb), over 200x coverage for the primary assembly. Additionally, we obtained 4,166,901 Illumina short reads (1.2 Gb) for polishing. The nanopore reads were split into four subsets to construct 20 independent *s*Gff assemblies that were merged into a consensus sequence using Autocycler and subsequently polished with the Illumina data. This approach produced a closed, circular 1.489 Mb reference genome from the *in vitro* cultured *s*Gff strain, designated *s*Gff-IAEA-CC, which was deposited in NCBI (RefSeq: GCF_049669535.1, BioProject: PRJNA1235259). We also assembled two additional *s*Gff genomes using publicly available NCBI SRA datasets from *Gff* flies (Bioproject: PRJNA1231403): one from a female IAEA colony fly (*s*Gff-IAEA-Fly) and another from a wild-caught female from northwestern Uganda (*s*Gff-UG-Tol).

For all assemblies, BUSCO scores exceeded 97.3% based on the OrthoDB entomoplasmatales_odb10 dataset, indicating high quality, complete genomes (Table 1). Genome size varied slightly among the assemblies. The *s*Gff-IAEA-CC reference genome was 1.489 Mb and included four plasmids ranging in size from 6 kb to 16 kb, while the *s*Gff-IAEA-Fly genome had a slightly smaller chromosome of 1.469 Mb (approximately 20 kb less than the reference *s*Gff-IAEA-CC genome), but retained all four plasmids. Interestingly, the *s*Gff-UG-Tol genome was more reduced, with a 1.418 Mb chromosome, and only three plasmids, missing the 13 kb plasmid, p_sGff2 (Table 1). We manually verified the absence of the plasmid p_sGff2 in *s*Gff-UG-Tol by mapping its reads to the *s*Gff-IAEA-CC assembly, confirming no coverage over the *s*Gff-IAEA-CC p_sGff2 region. Gene counts across assemblies showed moderate variation. The *s*Gff-IAEA-CC genome, including plasmids, contained 1,829 genes, including 1,687 protein-coding genes, 104 pseudogenes, 32 tRNAs, 3 rRNAs, and 3 non-coding RNAs (ncRNAs) (Table 1). In comparison, *s*Gff-IAEA-Fly encoded 1,795 genes (1,653 protein-coding genes), while *s*Gff-UG-Tol encoded 1,695 genes (1,553 protein-coding genes) (Table 1).

### *s*Gff clusters within the *Spiroplasma poulsonii* clade

**Figure 2:**
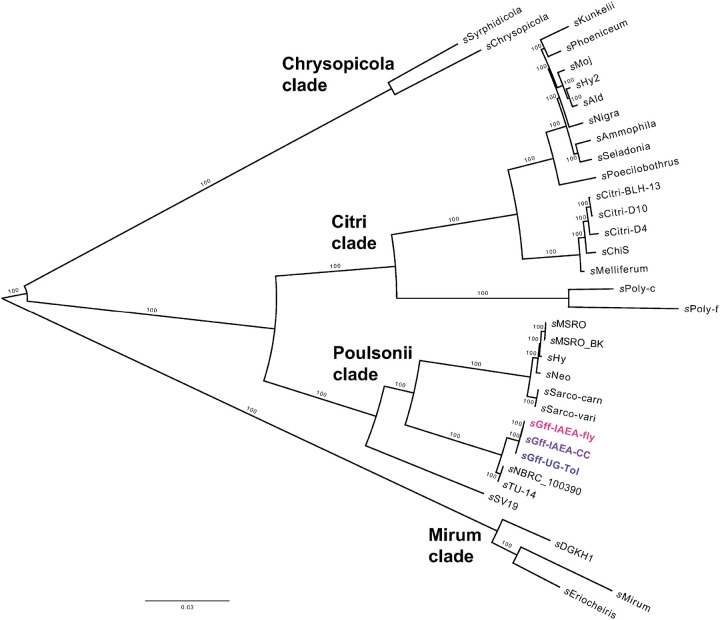
Phylogenetic placement of *s*Gff among representative *Spiroplasma* genomes. Maximum likelihood (RAxML) tree showing the relationship between *s*Gff and representative *Spiroplasma* species from the *Spiroplasma citri*, *Spiroplasma mirum, Spiroplasma chrysopicola,* and *Spiroplasma poulsonii* clades (see Supplementary Table 4 for accession details). Bootstrap support values are indicated on branches and the tree is midpoint rooted.

To place the newly assembled *s*Gff genomes within a broader phylogenetic framework, we constructed a RAxML maximum likelihood tree based on 93–101 single-copy core orthologous genes conserved in over 95% of representative *Spiroplasma* taxa from the *S. citri*, *S. mirum, S. chrysopicola,* and *S. poulsonii* clades (Figure 2). The *s*Gff isolates are grouped most closely with two poorly characterized but genetically identical *Spiroplasma* strains: *s*TU-14 and *s*NBRC_100390. The *s*TU-14 strain was originally isolated from a contaminated sample of another Mollicute (*Entomoplasma lucivorax* PIPN-2) and has an unknown host origin [92]. The *s*NBRC_100390 strain was initially misclassified as *Spiroplasma atrichopogonis* GNAT3597, a symbiont of biting midges, but has since been recognized as a distinct *Spiroplasma* taxon [93]. Together, the *s*Gff isolates, *s*TU-14, and *s*NBRC_100390 form a distinct, well-supported lineage within the *S. poulsonii* clade (Figure 2). To further resolve their evolutionary relationships, we constructed a higher-resolution RAxML phylogeny using 509–563 single-copy core orthologs shared among >95% of representatives of *Spiroplasma* taxa within the *Poulsonii* clade. This second RAxML tree reinforced the same relationships observed in the previous tree. It also resolved the *s*Gff polytomy, showing that the *s*Gff-IAEA-CC and *s*Gff-IAEA-Fly isolates were genetically identical and formed a monophyletic group, with *s*Gff-UG-Tol positioned as their sister lineage (Supplementary Figure 2).

### *s*Gff genomes are highly similar

**Figure 3:**
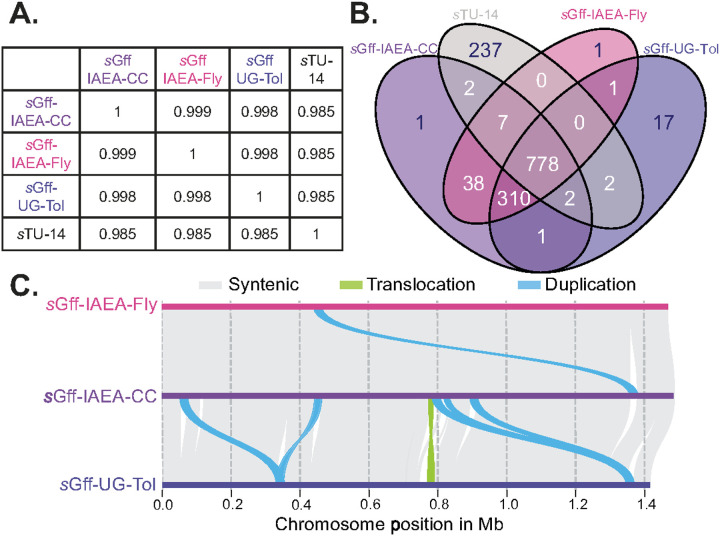
Genome comparisons between *s*Gff isolates and their closest relative, *s*TU-14. A) Average Nucleotide Identity (ANI) values between *s*Gff isolates and *s*TU-14, indicating high sequence similarity. B) Venn diagram showing shared and unique gene families for *s*Gff isolates and *s*TU-14. C) Genome synteny plot illustrating conserved gene order among *s*Gff isolates, highlighting structural similarities and isolate-specific variations.

Pairwise genome comparisons among the *s*Gff assemblies supported the relationships inferred from the RAxML phylogenies. Average Nucleotide Identity (ANI) was 99.9% between the *s*Gff-IAEA-CC and *s*Gff-IAEA-Fly genomes, and slightly reduced at 99.8% between the *s*Gff-IAEA-CC and *s*Gff-UG-Tol (Figure 3A). These ANI values are a magnitude higher than those observed between any *s*Gff isolate and its closest *Spiroplasma* outgroup, strain sTU-14, with pairwise ANI values of 98.5% to *s*Gff-IAEA-CC, *s*Gff-IAEA-Fly, and *s*Gff-UG-Tol (Figure 3A).

Gene content analysis revealed subtle differences between the nearly identical *s*Gff-IAEA isolates and the more divergent *s*Gff-UG-Tol isolate. Specifically, 38 gene families were unique to the *s*Gff-IAEA isolates, while 17 were unique to the *s*Gff-UG-Tol (Figure 3B). Among the 38 *s*Gff-IAEA-specific gene families, 20 were associated with mobile genetic elements (MGEs) – including twelve from plasmids with six from p_sGff2 that is absent from *s*Gff-UG-Tol, nine were related to metabolism and symbiosis, and the remaining nine encoded hypothetical proteins (Supplementary Table 10). The 17 *s*Gff-UG-Tol-specific gene families included four genes located on plasmid p_sGff3, three associated with MGEs, two symbiosis-related genes, and eight hypothetical proteins (Supplementary Table 11). When compared to the nearest sister taxon, *s*TU-14, we identified 310 unique gene families shared among the *s*Gff isolates, consisting mostly of MGEs, including *Spiroplasma* prophages, plasmids, and integrative conjugative elements (ICEs) (Supplementary Table 12).

Whole genome alignments further revealed that the two IAEA-derived *s*Gff genomes, *s*Gff-IAEA-CC and *s*Gff-IAEA-Fly, are nearly identical, differing by only three SNPs, 49 insertions/deletions (indels), and one duplication (Figure 3C and Supplementary Table 13). The three SNPs were located in genes encoding a prophage, the DNA-binding protein *WhiA*, and the fructoselysine transporter *frlA* (Supplementary Table 14). With no fructose supplemented in the culture medium, the nonsynonymous SNP converting threonine to arginine in *frlA* could be the result of relaxed selection. Most indels were located within homopolymeric tracts, suggesting potential assembly artifacts. However, we identified a notable 20 kb duplication involving a *Spiroplasma* prophage region in the *s*Gff-IAEA-CC genome, but absent compared to *s*Gff-IAEA-Fly (Figure 3C and Supplementary Table 15). This duplication likely represents a prophage polymorphism in the *s*Gff-IAEA isolate that became fixed during the *in vitro* culture process. In contrast, the *s*Gff-UG-Tol genome exhibited more extensive divergence, with 550 SNPs, 171 indels, five large duplications, and one translocation relative to *s*Gff-IAEA-CC (Figure 3C and Supplementary Table 16). Of the 550 SNPs, 383 were found within protein coding genes with over 44% associated with MGEs (Supplementary Table 17). While most of the indels were homopolymeric, at least 22 appeared to be genuine sequence differences rather than homopolymeric assembly artifacts. Large-scale structural variation between the *s*Gff-IAEA-CC and the *s*Gff-UG-Tol genomes included five duplications and one translocation (Figure 3C). All of these rearrangements were associated with *Spiroplasma* prophages (Supplementary Table 18).

### sGff mobile genetic elements

**Figure 4:**
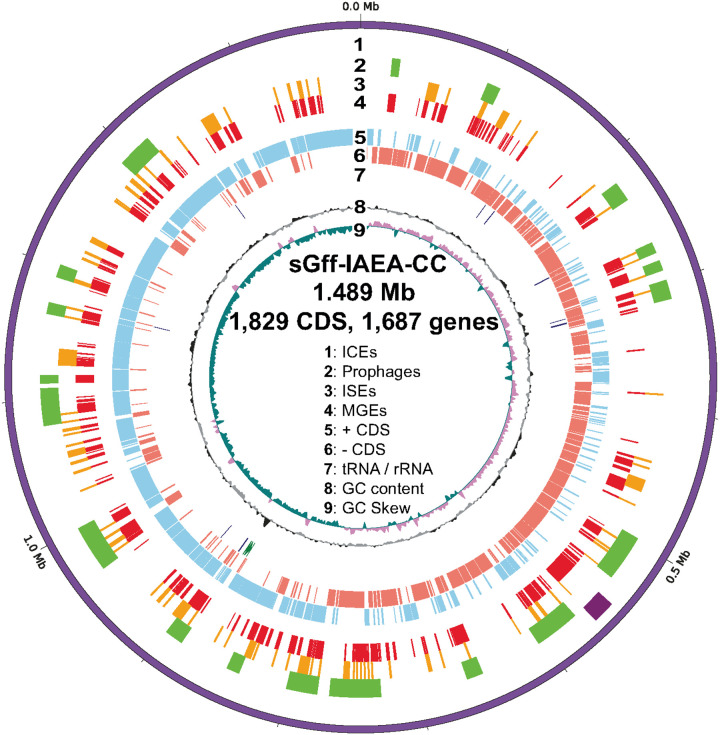
Circular genome plot of *s*Gff-IAEA-CC showing the distribution of Mobile Genetic Elements (MGE). The genome is represented in nine concentric rings (1–9), each corresponding to a feature labeled in the center of the plot. The figure highlights the high proportion and broad distribution of MGE-associated genes across the chromosome.

The *s*Gff-IAEA-CC chromosome encodes a total of 1,778 genes, including 1,637 protein-coding genes, of which 800 are predicted to encode hypothetical proteins with unknown functions. In addition, the genome contains 103 pseudogenes, 32 tRNAs, 3 rRNAs, and 3 non-coding RNAs (ncRNAs) (Table 1, Supplementary table 19). Genes associated with MGEs represented the most prevalent Cluster of Orthologous Groups (COG) functional category, accounting for over 28% of the annotated functions (Figure 4 and Supplementary Figure 3). When the 646 proteins with unknown COG annotations (36.33% of the genome) are excluded, the relative proportion of MGE-associated functions increases to 44.35%, highlighting the central role of MGEs in shaping the *s*Gff genome. Other prominent COG functional categories include translation, ribosomal structure and biogenesis, replication, recombination and repair, and carbohydrate transport and metabolism (Supplementary Figure 3).

The most common MGEs are *Spiroplasma* prophages, specifically from two families: Plectroviridae (*Plectovirus* prophages) and Microviridae (*Spiromicrovirus* prophages). Using Phastest, we identified 20 supported prophage regions, along with several other putative prophage-related genes scattered across the genome, including within plasmids (Figure 4). Insertion sequence (IS) elements were also highly prevalent, with 129 identified on the chromosome and many associated with prophage regions. In addition, a single well-supported ICE was identified that encoded a complete Type I restriction-modification system (Figure 4). Like the prophages, ICE-associated genes were dispersed throughout the genome, including some within prophage regions and plasmids.

### *s*Gff metabolism

We reconstructed the metabolic pathways of *s*Gff using PGAP-predicted proteins using BLASTKoala and eggNOG-mapper. This analysis revealed that *s*Gff possesses highly reduced biosynthetic capability and relies primarily on its *Gff* host for energy and essential metabolites (Supplementary Table 19). The bacterium’s energy metabolism centers on glycolysis, with glucose and fructose serving as its main carbohydrate substrates. We identified four phosphotransferase system (PTS) transporters predicted to facilitate uptake of specific sugars: fructose, glucose, 2-(alpha-D-mannosyl)-D-glycerate, and cellobiose/diacetylchitobiose. Notably, the presence of a diacetylchitobiose transporter, together with putative chitinases, suggests that *s*Gff may have the capacity to utilize chitin as an alternative energy source. The genome also reveals metabolic versatility through two additional pathways: a functional acetyltransferase-acetate kinase pathway indicating potential acetogenic metabolism, as well as geneclusters involved with sulfur reduction.

Like other *Spiroplasma* species, *s*Gff exhibits minimal capacity for *de novo* lipid synthesis, possessing functionality limited to the citric acid cycle and fatty acid elongation. Instead, it predominantly incorporates host-derived fatty acids and cholesterol directly into its cell membranes [94]. Nevertheless, *s*Gff retains crucial lipid-modifying capabilities, particularly the complete cardiolipin biosynthesis pathway that converts host-derived diacylglycerols (DAGs) into cardiolipin, a process identical to the experimentally verified pathway in *S. poulsonii* [95] that is essential for bacterial membrane formation [96]. The genome also encodes the non-mevalonate pathway for terpenoid backbone biosynthesis, providing additional lipid processing capability.

Beyond lipid modification, *s*Gff possesses several other significant biosynthetic capabilities, including a complete folate biosynthesis pathway, supporting one-carbon metabolism essential for nucleotide and amino acid synthesis. Hemolysin-related proteins and ferritin are also produced by *s*Gff potentially facilitating iron sequestration in the host environment (Supplementary Table 19). We also note that *s*Gff exhibits limited amino acid biosynthesis as it can only produce three amino acids *de novo*: aspartic acid, serine, and asparagine, indicating a high degree of dependence on host-derived nutrients.

### Symbiosis genes

In addition to its highly specialized metabolism, *s*Gff has several mechanisms that may facilitate its persistence within the tsetse host. Among these are lipoproteins, which play essential roles in symbiosis through interactions with the host immune system, host cells, and metabolites present in the hemolymph. Of the 1,637 predicted protein-coding genes in the *s*Gff genome, 72 encode lipoproteins (Supplementary Table 19). Many of these lipoproteins are associated with *Spiroplasma* prophage regions, suggesting that horizontal gene transfer and the utilization of prophage-derived lipoproteins may be key to *s*Gff’s adaptation to the host environment. The most abundant *Spiroplasma* lipoprotein is *Spiralin,* which is a well-characterized protein important in host interactions and vertical transmission [97]. We identified one conserved *Spiralin* gene (WP_424526001.1) as well as two other *Spiralin-*derived copies containing one and two repeats, respectively (WP_424525940.1 and WP_424525799.1). We note six adhesion-related genes: (WP_424527554.1, WP_424527500.1, WP_424527571.1, WP_424527528.1, WP_424527276.1, and WP_424526807.1) four of which are located on plasmids (one per plasmid) and are likely involved in host cell adhesion and invasion. Similar adhesion genes are essential for midgut cell invasion in bees [98] and adhesion to leafhopper cells [99].

We identified one putative ribosome-inactivating protein (RIP) gene in the *s*Gff genome (Supplementary Table 19). The gene (WP_424526995.1) encodes a protein with a conserved RIP domain, most similar to *s*Gff’s sister taxa *s*NBRC_100390 (Supplementary Figure 4). However, the *s*Gff RIP lacks a signal peptide, suggesting it is not secreted. Instead, this protein is predicted to be embedded in the outer cell membrane, with the RIP domain exposed to the intracellular environment. Whether this toxin is released via proteolysis by an unidentified peptidase remains unknown. We also identified a more divergent RIP-like protein (WP_424526987.1) that includes a signal peptide, suggesting it may be secreted into the host environment.

We identified two overlapping copies of the *glycerol-3-phosphatase* (*glpO*) gene in the *s*Gff genome (WP_424526233.1 and WP_424526234.1) (Supplementary Table 19). *glpO* catalyzes the oxidation of glycerol-3-phosphate, generating reactive oxygen species (ROS) as a byproduct of glycerol metabolism. In *Mycoplasma*, the gene functions as a virulence factor [100] and in *Spiroplasma* it may provide protection against parasitoid wasps [36]. Typically, *glpO* is part of a conserved operon flanked by *glpF* and *glpK*, which encode a glycerol transporter and glycerol kinase, respectively. However, in *s*Gff, *glpF* appears truncated due to the insertion of a *Spiroplasma* prophage, with only the first 32 amino acid residues present upstream of the insertion site. Interestingly, *in silico* analyses suggest that the truncated *glpF* and the two *glpO* copies remain potentially functional.

### Transcriptome validation

We performed RNAseq-based transcriptomic analysis utilizing three *in vitro* samples derived from *s*Gff culture and two *in vivo s*Gff samples isolated from *Gff* hemolymph. The transcriptomic dataset was first used to validate *in silico* annotations of the *s*Gff genome. We pooled transcripts from both culture and hemolymph samples, resulting in over 57 million reads mapped to the reference genome. Of the 1,778 annotated genes, 464 showed no detectable expression (TPM < 5) in either condition (Supplementary Table 20). Over 261 of these non-expressed genes were related to MGEs, primarily *Spiroplasma* prophages and suggests that prophage expression may be actively suppressed in *s*Gff. Among the 464 non-expressed genes, 52 were annotated as pseudogenes via *in silico* predictions. Interestingly, 52 of the 104 predicted pseudogenes exhibited transcriptional activity, with 34 annotated as MGEs, including transposases, phage-related proteins, and ICEs, indicating that some may retain regulatory or functional roles, particularly in genome plasticity and host adaptation. Additionally, we confirmed expression for all candidate symbiosis-associated genes identified in our genome analyses (Supplementary Table 20). Transcripts were detected from all four plasmids, indicating that plasmid-encoded genes are expressed under both *in vivo* and *in vitro* conditions.

We identified the most abundant transcripts from the pooled dataset. Among the top 20 most abundant genes, seven were associated with plasmid functions, three were involved in fructose metabolism and five were involved in core transcription and translation processes. The remaining highly expressed genes included two with unknown functions, a malate permease, a rod shape determining protein, and *Spiralin* (Supplementary Table 20). In addition, we used transcriptomic data to predict operon structures in the *s*Gff genome, as operons may encode co-regulated clusters, including putative secreted effectors relevant to host interactions or trypanocidal effects. Of the 1,675 protein-coding genes analyzed, 1,471 (87.8%) were predicted to be organized into 297 operons. The mean operon length was 3.95 genes, with the largest operon containing 39 genes (Supplementary Table 21). Operon 64 was particularly interesting as it encoded the conserved RIP, MATE family efflux transporters, and Peroxiredoxin, which is a strong antioxidant.

### Tissue dependent expression of sGff

**Figure 5:**
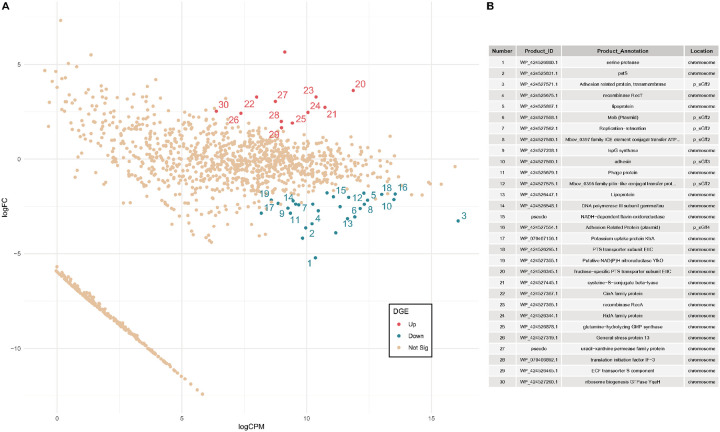
Differential gene expression between culture- and hemolymph-derived *s*Gff. A) MA plot depicting differentially expressed genes (DEGs) and their expression levels, with positive log-fold change values representing genes up-regulated in hemolymph-derived *s*Gff relative to culture-derived *s*Gff, and negative log-fold change values representing down-regulation. Genes were considered differentially expressed if they had a log_2_-fold change > 1.5 with FDR < 0.05 (Supplementary Table 22). Labeled genes correspond to those highlighted in panel B. B) Functional annotations and genomic locations (chromosome or plasmid) of the DEGs shown in panel A.

We next investigated tissue-dependent gene expression differences by comparing transcriptomes of *s*Gff isolated from hemolymph and from *in vitro* culture. In total, we identified 45 differentially expressed genes between the two sample types, using log_2_ fold change threshold > 1.5 or < −1.5 and a false discovery rate (FDR) < 0.05 (Figure 5A). Of these, 12 genes were upregulated in *s*Gff from hemolymph relative to the culture, while 33 genes were downregulated (Figure 5B and Supplementary Table 22). Among the 12 upregulated genes in from hemolymph relative to culture, 11 were located on the main chromosome, and one was plasmid-encoded. The upregulated main chromosomal genes contained five metabolism-related genes, including a fructose-specific phosphotransferase system (PTS) transporter, two translation-related genes, a detoxifying-related gene, a repair-related gene, a stress-related gene, and a pseudogene. Of the 33 downregulated in *s*Gff hemolymph compared to culture, 21 were located on the main chromosome and 12 were plasmid-encoded. The genes on the chromosome included seven hypothetical proteins, six metabolism-related genes, including a glucose-specific PTS transporter, six prophage-related genes, two lipoproteins, a DNA polymerase, and a serine protease.

These differential expression patterns likely reflect environmental differences between the *in vivo* (hemolymph) and *in vitro* (culture) conditions. The upregulation of a fructose-specific PTS transporter and downregulation of a glucose-specific PTS transporter may indicate a shift in available carbohydrate sources between the hemolymph and the culture medium, as fructose is absent from the culture media (Supplementary Document 1). Reduced expression of prophage genes could also suggest *s*Gff can suppress prophage expression in certain environments.

## Discussion

This study advances our understanding of the interactions between *Glossina fuscipes fuscipes* (*Gff*) and its symbiont *Spiroplasma glossinidia* (*s*Gff) through the establishment of an *in vitro* culture system, whole genome sequencing of multiple *s*Gff isolates, and transcriptomic comparisons between *s*Gff derived from culture and from *Gff* hemolymph.

While *Spiroplasma citri* was first cultured in 1971 [101] and some *Spiroplasma* strains were propagated using both cell-based and cell-free culture systems [102, 103], optimizing growth conditions for many strains remained challenging, likely due to the bacterium’s specialized adaptations to its specific insect host environment [104]. More recently, the use of Barbour-Stoenner-Kelly (BSK-H) media, supplemented with specific nutrients, has facilitated the sustained *in vitro* growth of the previously unculturable *s*MSRO strain [39], and now *s*Gff. The initial growth pattern of cultured *s*Gff was characterized by two exponential phases separated by a plateau phase. Culture plateaus at specific density thresholds were also observed in *s*MSRO [105], however, the *sGff* culture resumed growth after the plateau phase, possibly reflecting adaptation to the culture environment or modulation of endogenous prophages, which can impact growth dynamics in related strains [106, 107]. The growth rate of *sGff* was slower than *s*MSRO, with a doubling time of 40 hours compared to 30 hours, which may reflect strain-specific metabolic traits [39]. Further optimization of the *s*Gff culture media, informed by insights from its genome-derived metabolic profile [104], may improve these growth rates. Importantly, *s*Gff retained capacity for infection after prolonged *in vitro* cultivation, as demonstrated by successful colonization of naïve *Gff* following experimental infection, with higher inoculum doses resulting in higher *s*Gff titers. It will be important to establish whether *s*Gff infections can recapitulate the same tissue tropism and transmission dynamics of natural infections. Overall, the availability of a reliable culture system provides a powerful tool for dissecting the molecular dialogue between *s*Gff and its tsetse host, including the potential for genetic manipulation of the symbiont.

Using a hybrid sequencing approach that combined Nanopore and Illumina reads, we assembled a high-quality, closed genome from the *s*Gff culture consisting of a 1.489 Mb chromosome along with four plasmids ranging in size from 6 to 16 kb. This reference genome (*s*Gff-IAEA-CC) was compared to two additional *s*Gff assemblies – one from a colony fly (*s*Gff-IAEA-Fly) and another from a fly collected in Northwest Uganda (*s*Gff-UG-Tol). Comparative analysis confirmed that all three assemblies represent closely related isolates of the same strain, differing by several SNPs, indels, and unique gene families, with very little genomic variation between the sGff-IAEA-CC and sGff-IAEA-Fly genomes. The few genomic differences observed between the two IAEA-fly derived *s*Gff genomes could be attributed to either natural polymorphisms that became fixed during cultivation or perhaps from an elevated mutation rate due to the absence of genes in the mismatch repair pathway, namely *MutS*, *MutL*, and *MutH*, which is a trait shared across the *Spiroplasma* genus [35]. In contrast, more pronounced genomic variation was observed between the IAEA-derived genomes and field-derived *s*Gff-UG-Tol isolate. This divergence is consistent with the moderate genetic differentiation between their respective *Gff* hosts, as *s*Gff-UG-Tol isolate was obtained from a Northwestern Ugandan population, while the IAEA *Gff* colony originated from flies collected in the Central African Republic in 1986 [47, 108]. Nearly all the genomic variation between these isolates was localized to MGEs, including genes associated with *Spiroplasma* prophages. Notably, one plasmid present in the IAEA isolates was absent in the Uganda field isolate. Studies characterizing the range of genetic variation found in *s*Gff across the landscape will be important for understanding the full extent of genetic diversity in this symbiont.

Phylogenetic analysis placed the *s*Gff isolates within the *S. poulsonii* clade, a group that contains several other well-characterized *Spiroplasma* strains known for protecting their hosts against nematodes, parasitoids, and viruses [109–111]. Among these sister strains is *s*MSRO, whose culture protocol we successfully adopted for *s*Gff and likely explains the ease with which *s*Gff was cultivated. The closest known relatives of *s*Gff are two genetically identical but poorly characterized strains: *s*TU-14 and *s*NBRC_100390. These strains share 98.5% genome-wide identity with *s*Gff and have 778 gene families in common. However, *s*Gff possesses over 310 unique gene families, consisting primarily of hypothetical proteins and MGEs. These shared and unique gene families highlight both its close relationship to its sister taxa as well as its substantial repertoire of distinctive genetic features.

The *s*Gff genome consists of a remarkably high proportion of MGEs, including prophages, integrative conjugative elements (ICEs), and insertion sequence elements (ISEs), which together comprise over 28% of the genome. When hypothetical proteins are excluded, MGE-associated content increases to more than 44% of the annotated genome. This high MGE load is consistent with observations in several other *S. poulsonii* and *S. citri* strains [35, 112]. In total, we identified 20 distinct prophage regions, consisting largely of *Spiromicrovirus* and *Plectrovirus* prophages, along with one ICE, and 129 ISEs. We also identified additional prophage- and ICE-related genes dispersed throughout the genome and plasmids – all pointing to a dynamic genome characterized by extensive structural and functional plasticity. Prophages and MGEs can play major roles in the evolution and virulence of their hosts [113–116], acting as a reservoir of adaptive genes that can either be co-opted or horizontally transferred to enhance symbiotic capabilities [116, 117]. For example, in *Wolbachia*, key symbiosis-associated genes, such as the *cif* genes responsible for cytoplasmic incompatibility [118], and *wmk*, a male-killing factor [119], are both derived from and horizontally transferred via prophages [120, 121]. In *s*Gff, as well as other *Spiroplasma* strains, this pattern holds true [35, 122]. In *s*Gff, key symbiosis genes, such as *RIP* and *glpO*, are flanked by prophage sequences. Several prophages in *s*Gff encode lipoproteins, which could mediate interactions with host cells, metabolites, and lipids, contributing to symbiotic function [123–125].

The abundance of prophages present in the *s*Gff genome may also play a mechanistic role in regulating *Spiroplasma* symbiosis. In *Wolbachia*, the genomic island *octomom* modulates bacterial density to prevent overproliferation [126]. Although *s*Gff lacks a clearly defined *octomom* ortholog, prophage induction may serve a similar density-dependent regulatory function, by triggering bacterial lysis in response to stress, such as overcrowding or resource limitation, [127, 128] or via a quorum sensing mechanism as observed in *Vibrio cholerae* [129] or *E. coli* [130]. Our transcriptomic comparison between cultured *s*Gff and *s*Gff isolated from tsetse hemolymph supports this hypothesis, as prophage-associated genes were highly expressed in the dense *in vitro* cultures as compared to hemolymph-derived *s*Gff. Additionally, prophage induction can also modulate biofilm formation [131], interbacterial competition [132], or be induced via reactive oxygen species (ROS) [133]. Future studies comparing prophage activity in trypanosome-infected and uninfected tsetse will be key in determining whether *s*Gff prophages contribute to trypanosome resistance or influence interactions with other prominent tsetse symbionts, including *Sodalis* and *Wigglesworthia*.

Our metabolic profiling confirms that, like other *Spiroplasma* strains [134, 135], *s*Gff has limited biosynthetic capacity and relies on its host for essential metabolites. Unlike its sister taxon *s*MSRO that utilizes only glucose [36], *s*Gff appears to use both fructose and glucose as primary carbon sources. Notably, a functional trehalose transporter was absent, which may reduce *s*Gff pathogenicity, as trehalose is abundant in tsetse [136]. The inability of sGff to utilize trehalose could limit its replication within the host and reduce host resource exploitation, thereby supporting a more commensal relationship. Transcriptomic analysis revealed condition-specific regulation of carbohydrate transporters, where fructose-specific transporters were upregulated in hemolymph-derived *s*Gff, while glucose-specific transporters were downregulated. This finding likely reflects differences in nutrient availability, as fructose is absent from the culture medium, and suggests that fructose supplementation may accelerate *in vitro* growth. Nutrient-driven tissue tropism may also influence *s*Gff distribution in its *Gff* host. High fructose levels in reproductive tissues [137] could explain the elevated *s*Gff titers observed in these organs, while post-blood meal glucose spikes [138] may promote gut colonization and proliferation. We also identified a transporter for diacetylchitobiose, a sugar derived from chitin. While *s*Gff encodes several putative chitinases, it may access chitin breakdown products indirectly, potentially through *Sodalis*-derived secreted chitinase enzymes [139].

Beyond carbohydrates, *s*Gff scavenges host-derived fatty acids and cholesterol, and like other *Spiroplasma* strains, can incorporate them directly into its membrane [94]. Pathway reconstructions also indicate that *s*Gff can metabolize host diacylglycerols (DAGs) to synthesize cardiolipins, which are key membrane lipids in *Spiroplasma* [94] that could be cytotoxic [36] and in *Drosophila*, serve as important virulence factors by depleting host DAGs [95]. In *Gff* flies, the lipid-depleting phenotype associated with *s*Gff is supported by previous work that shows decreased expression of *Gff* genes involved in fatty acid synthesis in *s*Gff infected flies, suggesting reduced lipid availability [16]. In addition, triacylglycerol (TAG) levels are decreased in the fat bodies of *s*Gff-infected tsetse [17] and since TAGs are synthesized from DAGs, sGff’s consumption of host DAGs may be contributing to this decrease. Functional annotations also indicated that *s*Gff can produce ferritin and a hemolysin-related protein, both of which are used for iron sequestration. Iron sequestration is an important aspect of nutritional immunity, limiting microbe overproliferation and providing pathogen resistance [111, 140, 141] and In *Drosophila*, the iron-binding protein transferrin is upregulated in the presence of *s*MSRO, which relies on host transferrin-bound iron for survival [142]. Finally, consistent with other *Spiroplasma* species, *s*Gff is unable to synthesize most amino acids, and instead must import them from the host, further emphasizing its nutritional dependence on *Gff* [97, 143].

Our metabolic analysis reveals an intriguing potential competitive dynamic between *s*Gff and trypanosomes within the tsetse gut, as both microorganisms require the same host-derived resources. In the *Gff* gut, both organisms prefer glucose as their primary carbohydrate source, with trypanosomes later switching to proline [138] – an amino acid that *s*Gff also catabolizes. Trypanosomes, like *s*Gff, scavenge host lipids and cholesterol [144] and both reduce the expression of *Gff* genes associated with lipid biosynthesis [16] and deplete host TAG reserves [17, 145]. While trypanosomes acquire transferrin-bound iron through a high affinity receptor [146], *s*Gff likely acquires transferrin-bound iron in a manner similar to its sister *s*MSRO strain [142]. This potential multifaceted resource competition may contribute to the trypanosome-refractory phenotype observed in *s*Gff-infected flies [18]. By limiting parasite access to essential nutrients, *s*Gff may delay parasite proliferation, potentially providing the host sufficient time to mount an effective immune response. These competitive dynamics require new studies to characterize sGff’s metabolic demands *in vivo* and to test how resource competition influences host-pathogen dynamics, perhaps contributing to trypanosome resistance in tsetse.

In addition to a nutritional impact, *Spiroplasma* produces a diverse repertoire of toxins that can impact host fitness via reproductive manipulation or protection against parasites and pathogens [109, 110]. In *s*Gff, we identified a limited set of potential encoded toxins: two putative Glycerol-3-phosphatase (*glpO*) genes and one ribosome-inactivating-protein (RIP) gene – all of which are expressed according to our transcriptome data. The *glpO* enzyme generates reactive oxygen species (ROS) and is a major virulence factor in *Mycoplasma* [147] and may protect *s*MSRO-infected *Drosophila* against parasitoids [36]. In response to trypanosome infection in the gut, tsetse express nitric-oxide synthase (NOS), which catalyzes the production of trypanocidal nitric oxide (NO) and ROS [16]. These ROS are an essential part of the tsetse innate immune response, such that supplementing infected blood meals with antioxidants significantly enhances trypanosome infection success [148]. Thus, the ROS produced by *glpO* encoded by *s*Gff could contribute to *Gff’s* immune response and enhance its trypanocidal effect.

We identified one well-conserved RIP gene in the *s*Gff genome, which is transcriptionally active. RIPs act by depurinating the sarcin-ricin loop of 28s rRNA, thereby damaging host ribosomes and causing cell-death [149]. In sister *S. poulsonii* strains, *s*MSRO, *s*HYD, and *s*NEO, RIPs can protect their hosts from parasitic nematodes and parasitoid wasps [28, 29]. Unlike most RIP genes present in the *S. poulsonii* clade, which are typically secreted, the *s*Gff RIP gene lacks a signal peptide, [109] and is predicted to be anchored in the cell membrane. In dense microenvironments such as plaques, high concentration of the membrane-bound RIPs could exert toxic effects in close proximity, while having limited effects at a distance. Alternatively, the RIP may be proteolytically cleaved from the membrane and released into the extracellular space, enabling it to act on specific targets, or perhaps secreted by MATE family efflux transporters encoded within the same operon. Given that secreted RIPs exhibit high substrate specificity, future work is needed to determine the nature of its molecular targets and whether this RIP remains membrane-associated or is secreted. Furthermore, other potential RIP functions need to be evaluated, including enzymatic activity such as chitinase or phosphatase activity on lipids [150]. RIPs can also impact host tissues and incur fitness costs [28], but such effects may be tolerated or advantageous in contexts where protection against parasites or pathogens improves host survival. This trade-off could explain the polymorphic *s*Gff distribution in *Gff* populations in northern Uganda, where it persists at about ~ 30% prevalence [18]. The potential for RIPs to mediate protective benefits in a populationspecific manner warrants future research, particularly to determine whether *s*Gff-derived RIPs are active against trypanosomes.

## Conclusions

Here, we report the first successful *in vitro* cultivation and complete genome assembly of the *Spiroplasma glossinidia* strain *s*Gff — the *Spiroplasma* symbiont of *Gff* associated with deleterious effects on host metabolism and a trypanosome-refractory phenotype. We assembled a closed, circular ~1.5 Mb genome that clusters within the *S. poulsonii* clade. Comparative genomic analyses of cultured, laboratory- and field-derived *s*Gff isolates revealed only minor isolate-specific genetic variations, residing primarily in mobile genetic elements in field-derived *s*Gff. Metabolic profiling confirmed that *s*Gff has limited biosynthetic capacity and relies on its *Gff* host for essential carbohydrates, lipids, and amino acids. We also identified two putative toxin genes: *RIP* and *glpO* that may contribute to its trypanocidal potential. Our genomic discoveries and the availability of a stable culture system will enable future functional studies to elucidate this symbiosis and identify potential implications for trypanosome transmission control.

Symbionts can protect their hosts through several mechanisms, including immune priming, nutrient supplementation, competitive exclusion of pathogens, or secretion of effector proteins that target pathogens. In the case of *s*Gff, previous work found no evidence for activation of the tsetse immune system [16], and our work here identified no key nutrient supplementation apart from folate, which is already produced in excess by *Gff’s* obligate symbiont *Wigglesworthia*. However, we identified several points of potential metabolic competition between *s*Gff and trypanosomes, including glucose, cholesterol, fatty acids and iron, which could collectively have an additive effect to limit parasite fitness. In addition, the two toxin genes identified, *glpO* and *RIP,* represent potential candidates for direct trypanocidal activity. These genomic discoveries, along with the establishment of a stable culture system, lay the foundation for functional studies to dissect the mechanisms of trypanosome resistance. Such studies may involve metabolomics, RNAseq, culture media modifications, tsetse transfections, or perhaps *s*Gff gene knockouts. Future work also needs to address fundamental aspects of *s*Gff biology, including its tissue specific distribution, specific roles of *s*Gff within different host compartments, and strategies it uses for vertical transmission. Expanding transcriptomic analyses to additional tissues, such as the reproductive organs, salivary glands, and midgut, will help reveal whether *s*Gff gene expression is spatially regulated and potentially linked to the diverse physiological phenotypes observed in the tsetse host.

## Supplementary Files

This is a list of supplementary files associated with this preprint. Click to download.

- SupplementaryDocument1.pdf
- SupplementaryTables122.xlsx
- SupplementaryFigures.docx

## Figures and Tables

**Table 1.** Assembly statistics for the three *s*Gff genomes.

	sGff-IAEA-CC	sGff-IAEA-Fly	sGff-UG-Tol
**Chromosome (bp)**	1,489,281	1,469,171	1,418,152
**p_sGff1 (bp)**	16,389	16,389	16,375
**p_sGff2 (bp)**	13,621	13,621	N/A
**p_sGff3 (bp)**	13,208	13,208	12,717
**p_sGff4 (bp)**	6,466	6,466	6,460
**GC content**	27.16%	27.21%	27.29%
**BioProject**	PRJNA1235259	PRJNA1235259	PRJNA1235259
**BioSample**	SAMN47572768	SAMN48057343	SAMN48057344
**Assembly**	GCF_049669535.1	GCA_049949145.1	GCA_049949155.1
**BUSCO score**	C:97.6% [S:97.6%,D:0.0%]	C:97.6% [S:97.6%,D:0.0%]	C:97.3% [S:97.3%,D:0.0%]
**genes**	1,829	1,795	1,695
**protein-coding genes**	1,687	1,653	1,553
**pseudogenes**	104	104	104
**tRNA**	32	32	32
**rRNA**	3	3	3
**ncRNA**	3	3	3

BUSCO scores are for the entomoplasmatales_odb10 OrthoDB dataset

## Data Availability

All genomic and transcriptomic data generated in this study is available at the NCBI BioProject: PRJNA1235259. Raw data from culture growth kinetics and injection experiments is deposited under https://doi.org/10.60600/YU/2979VU.

## Abbreviations

AAT: African Animal Trypanosomiasis
ANI: Average Nucleotide Identity
BLASTP: Basic Local Alignment Search Tool for proteins
BSK-H medium: Barbour-Stoenner-Kelly H medium
BUSCO: Benchmarking Universal Single-Copy Orthologs
CDS: Coding Sequences
COG: Cluster of Orthologous Groups
DAGs: Diacylglycerols
DNA: Deoxyribonucleic acid
E. coli: Escherichia coli
FDR: False-Discovery Rate
GC content: Guanine-Cytosine content
Gff: Glossina fuscipes fuscipes
HAT: Human African Trypanosomiasis
HMW DNA: High Molecular Weight Deoxyribonucleic Acid
IAEA: International Atomic Energy Agency
ICEs: Integrative Conjugative Elements
ISEs: Insertion Sequence Elements
KEGG: Kyoto Encyclopedia of Genes and Genomes
MGEs: Mobile Genetic Elements
ncRNAs: non-coding Ribonucleic Acids
NCBI: National Center for Biotechnology Information
NCBI SRA: National Center for Biotechnology Information Sequence Read Archive
NO: Nitric Oxide
ONT: Oxford Nanopore Technologies
PCR: Polymerase Chain Reaction
p_sGff: Plasmid of *Spiroplasma* endosymbiont of *Glossina fuscipes fuscipes*
PTS transporters: Phosphotransferase System Transporter
qPCR: quantitative Polymerase Chain Reaction
RIPs: Ribosome-inactivating Proteins
RNA / rRNA: Ribonucleic Acid / ribosomal Ribonucleic Acid
RNAseq: Ribonucleic Acid Sequencing
ROS: Reactive Oxygen Species
*s*Gff: *Spiroplasma* endosymbiont of *Glossina fuscipes fuscipes* / strain *s*Gff
*s*MSRO: *Spiroplasma poulsonii* Melanogaster Sex-Ratio Organism
SNPs: Single Nucleotide Polymorphisms
*Sp*AID: *Spiroplasma poulsonii* Androcidin
TAG: Triacylglyceride
TPM: Transcripts per Million
tRNA: transfer Ribonucleic Acids
